# Spatio-temporal prevalence of porcine cysticercosis in Madagascar based on meat inspection

**DOI:** 10.1186/s13071-015-0975-2

**Published:** 2015-07-25

**Authors:** Vincent Porphyre, Harentsoaniaina Rasamoelina-Andriamanivo, Andriatsilavina Rakotoarimanana, Ony Rasamoelina, Claire Bernard, Ronan Jambou, Eric Cardinale

**Affiliations:** CIRAD, UMR112 SELMET, Saint Pierre, La Réunion France; FOFIFA-DRZV, Antananarivo, Madagascar; Faculty of Veterinary Medicine, University of Antananarivo, Antananarivo, Madagascar; CIRAD, UMR CMAEE, Ste Clotilde, La Réunion France; CRVOI, Ste Clotilde, La Réunion France; Institut Pasteur, Paris, France

**Keywords:** Cysticercosis, Pig, Epidemiology, Slaughterhouse, Meat inspection, Food safety, Madagascar

## Abstract

**Background:**

*Taenia solium* cysticercosis is a parasitic meat-borne disease that is highly prevalent in pigs and humans in Africa, but the burden is vastly underestimated due to the lack of official control along the pork commodity chain, which hampers long-term control policies.

**Methods:**

The apparent and corrected prevalences of *T. solium* cysticercosis were investigated in pork carcasses slaughtered and retailed in Antananarivo (Madagascar), thanks to a 12-month monitoring plan in two urban abattoirs.

**Results:**

Overall apparent prevalence was estimated at 4.6 % [4.2 – 5.0 %]. The corrected overall prevalence defined as the estimated prevalence after accounting for the sensitivity of meat inspection was 21.03 % [19.18- 22.87 %]. Significant differences among geoclimatic regions were observed only for indigenous pigs, with an apparent prevalence estimated at 7.9 % [6.0 – 9.9 %] in the northern and western regions, 7.3 % [6.0 – 8.6 %] in the central region, and 6.2 % [4.7 – 7.8 %] in the southern region. In the central region, where both exotic and indigenous pigs were surveyed, indigenous pigs were 8.5 times [6.7 – 10.7] more likely to be infected than exotic improved pigs. Urban consumers were more likely to encounter cysticercosis in pork in the rainy season, which is a major at risk period, in particular in December. Differences between abattoirs were also identified.

**Conclusion:**

Our results underline the need for improved surveillance and control programmes to limit *T. solium* cysticercosis in carcasses by introducing a risk-based meat inspection procedure that accounts for the origin and breed of the pigs, and the season.

## Background

The pork tapeworm *Taenia solium* continues to be a major cause of epilepsy in Africa and represents a heavy burden for the pork value chain. After ingesting the parasite’s eggs, pigs become infected and larvae form cysts in muscle tissue of the host (porcine cysticercosis); people who eat contaminated pork will then harbor an adult tapeworm in their intestine. At that stage, the tapeworm produces few or no symptoms (taeniasis), but when it expels its reproductive segments in human faeces, infective eggs are disseminated into the environment and/or can contaminate human food. In this case, a person who ingests infective eggs can develop the human cysticercosis phase of the disease, which causes chronic headaches, meningitis, blindness, and even death when the tapeworm’s larvae enter the brain and trigger severe epileptic seizures [1].

In Madagascar, some basic steps are still needed to assemble sufficient data to draw a clear picture of the taeniasis/cysticercosis complex. Human taeniasis is clearly underestimated but neurocysticercosis is frequently reported [2]. Studies carried out in Madagascar between 1994 and 1999 reported an antibody seroprevalence to human cysticercosis ranging from 7 % to 20 % [3] with the highest levels recorded in the central highlands and less than 10 % in coastal areas [4].

Pork contaminated by *T. solium* is expected to be highly prevalent due to the serious lack of sanitation infrastructure, to roaming pigs in villages, and/or to biosecurity breaches in pig farms [5], but the epidemiological situation is certainly underestimated. Official veterinary services reported an overall prevalence of porcine cysticercosis of around 0.5 to 1 % at the slaughterhouse [2], surprisingly suggesting that exposure by Malagasy consumers to infected meat could be low. Given the informal organization of the pig sector, estimating prevalence is a challenge, even though it is crucial to identify the geographical distribution of the parasite before planning control measures [6].

The aim of this study was to estimate the overall prevalence of *T. solium* cysticercosis in the swine population in the main pig production areas of Madagascar, and to investigate the main parameters associated with cysticercosis infection in pig carcasses in two urban abattoirs in Antananarivo, the capital.

## Methods

### Field data

A pilot surveillance system was implemented in the two main urban abattoirs among a total of five official slaughterhouses (comprising a total of 15 slaughtering slabs) located in Antananarivo (central Madagascar): the main slaughterhouse, Anosizato (Abattoir #1) had 11 registered slaughtering slabs, whereas Ankadintratombo (Abattoir #2) had only one. Each slaughterhouse was monitored daily over a period of 12 months from March 2013 to February 2014. Information was collected with the active participation of veterinary students from the Veterinary School (Antananarivo University), local veterinary officers, technicians in charge of official meat inspection and professionals in the pork value chain. In abattoir #1, an average of 172 pigs were recorded daily (for a total period of 346 days) by a veterinary student who benefited from the strong involvement of stakeholders: every day after the pigs were slaughtered and dressed, butchers and slaughterhouse workers gave each carcass a preliminary visual and incisional inspection, and informed our surveyor of the need for further examination when necessary. In abattoir #2, every day, a second veterinary student examined each animal slaughtered (daily average 23 animals) for a total period of 341 days. In both situations, the status of carcasses suspected of harboring cysticercosis was confirmed by incisional examination following the local meat inspection procedure [7, 8]. The heart, masseters, diaphragm, and tongue were examined by eye. Long parallel incisions were made in external and internal masseter muscles. The tongue was palpated and a longitudinal incision was made at the base of the tongue to look for cysts. The heart was cut open to detect cysts in the septum [9]. Information was recorded on the breed, day of slaughter, and region where the animal was raised. The cysticerci stages, i.e. viable or degenerated, were not recorded. No information was recorded on the number of larvae in the muscles or on the location of cysticerci lesions.

Urban markets in Antananarivo are supplied by a complex network of traders, and pigs from all over Madagascar are transported from distant regions and slaughtered in urban abattoirs [2]. Unfortunately, traceability of animals is illusory and the only reliable information for surveillance at the final market is likely to be the production area [10, 11]. For the purpose of this study, the administrative regions in Madagascar were aggregated into four meta-regions based on their geoclimatic characteristics: (i) the north and north-western region, which includes Diana and Sofia regions, has an equatorial climate with temperatures ranging from 15 °C to 37 °C; (ii) the western region, which comprises three regions (Boeny, Menabe, Melaky), has less rainfall, and temperatures range from 10 °C to 37 °C; (iii) the central region of Madagascar is made up of uplands (Analamanga, Amoron'i Mania, Bongolava, Vakinankaratra, Itasy, Haute Matsiatra, Ihorombe), the altitude ranges from 1200 to 1500 meters asl., and the climate resembles a Mediterranean climate (average temperature 20 °C); and (iv) the southern region, which includes Anosy, Antsimo Andrefana regions, is a dry sub-desert area with a wide range of temperatures (from 6 °C to 40 °C). The seasons were defined as follows: fall (cool dry season) from April to May, winter (cold dry season) from June to August, spring (warm dry season) from September to October, and summer (warm rainy season) from November to March.

### Data analysis

Apparent prevalence with a 95 % confidence interval (CI) was calculated using data from our sample survey dataset detailing the number of positive animals vs. negative animals, on each day of the survey, for each breed (improved vs. indigenous), for each slaughterhouse (n = 2) and for each production commune (n = 79). Apparent prevalence was defined as the number of pigs found to have cysts during meat inspection divided by the total number of pigs slaughtered.

Corrected prevalence was calculated by dividing apparent prevalence by the “detection fraction” [12] i.e. the proportion of infected pigs successfully detected under meat inspection procedures in African countries. The sensitivity and specificity for detecting *T. solium* cysticercosis by meat inspection in African conditions was previously calculated using a Bayesian approach to be 22.1 % and 100 % respectively [13]; in this particular situation where, given a specificity of 100 %, false positive results could be disregarded, the detected fraction was similar to the sensitivity and was estimated at 22.1 %.

Multivariate logistic regression analyses were performed to test the associations between risk factors (individual and group-level) with the observed prevalence of *T. solium* on carcasses as outcome variable. Taking into account overdispersion in the total dataset and missing values, three models were run to cover every combination of parameters: (i) model 1 compared prevalence only in indigenous pigs in the central, northern and western climatic regions because of the poor availability of information about commercial breed pigs; (ii) model 2 investigated prevalence in southern regions where observations were sparse, and (iii) model 3 explored prevalence in the administrative regions in the central upland climatic area, for which our dataset was sufficiently complete to assess the association of breed with cysticercosis infection.

The modeling selection strategy of Hosmer and Lemeshow was used [14]: the statistical significance of each variable was evaluated separately. Each variable with a *p-value* lower than 0.20 was included in a generalized linear model (GLM) for multivariate analysis. The contribution of each factor to the model was tested using a likelihood ratio *χ*^2^ in a stepwise procedure. The simpler model was determined using the lowest value of the Akaike information criterion (AIC) [15]. The goodness-of-fit (GOF) of the final model was assessed using Pearson *χ*^2^, deviance and Hosmer-Lemeshow tests [16]). Odds ratios were calculated to assess the contribution of each explanatory variable selected in the final model to *T. solium* contamination. Statistical analyses were performed using R software version 3.0.3 [17]. The apparent prevalence of cysticerci as a function of the region in Madagascar between March 2013 and February 2014 was mapped to evaluate the spatial distribution of prevalence using Quantum GIS software [18].

### Ethical considerations

At the time of the study, neither the Malagasy Department of Veterinary Research and Husbandry nor the Ministry of Livestock Production had a committee to review and approve scientific research protocols involving animals. However, no animals were euthanized for the purpose of the study since we only considered the *T. solium* cysticercosis status of animals routinely slaughtered in Malagasy slaughterhouses.

## Results

### Descriptive results

**Fig. 1:**
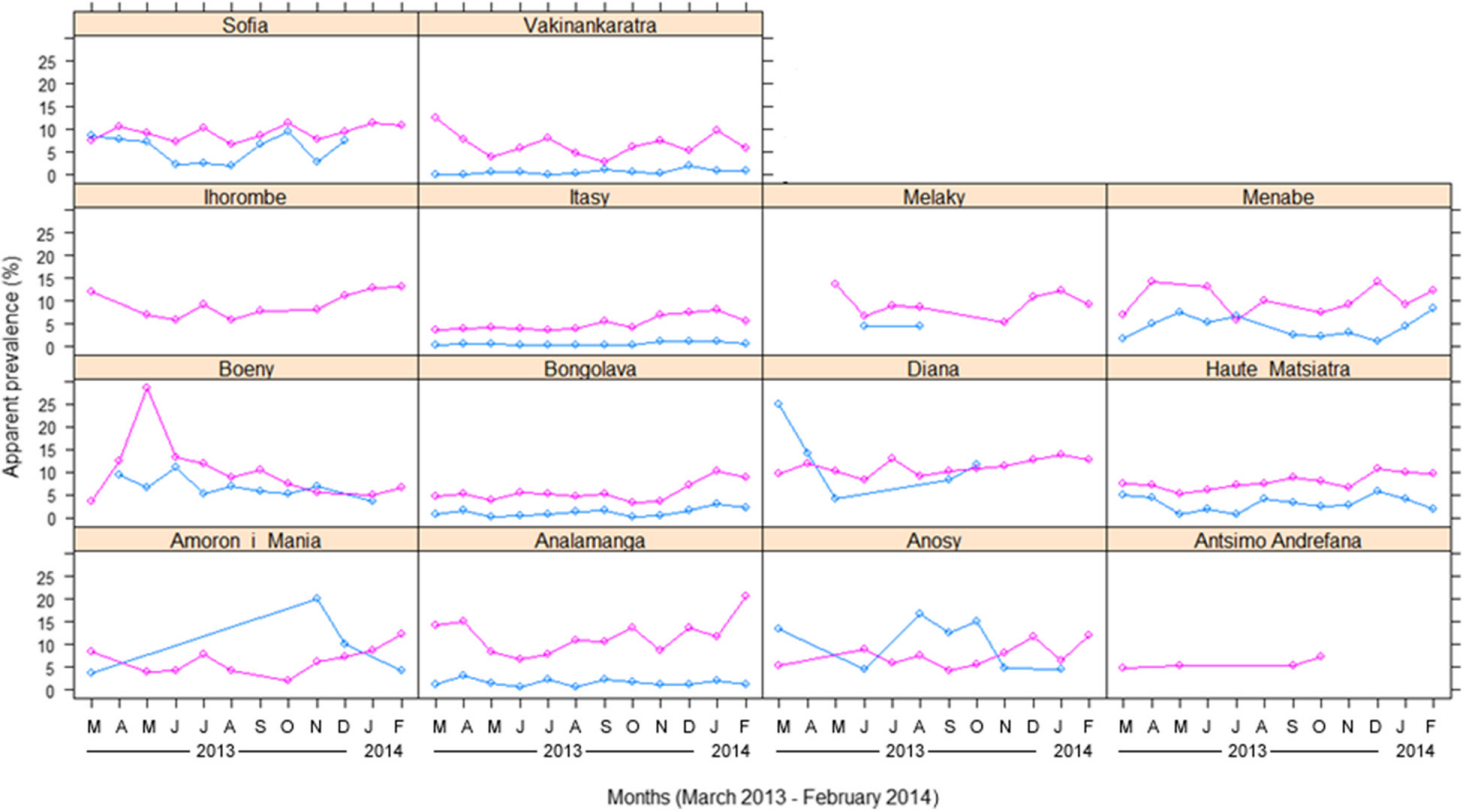
Monthly prevalence of cysticercosis positive carcasses of local breed (n = 41,178; pink line) and exotic breed (n = 27,254; blue line) pigs raised in 14 regions and slaughtered in 2 abattoirs in Antananarivo, Madagascar (March 2013 -February 2014)

From March 2013 to February 2014, a total of 68,432 pigs were slaughtered in the two abattoirs we surveyed, Anosizato (n = 59,765 carcasses, Abattoir #1) and Ankadintratombo (n = 8667, Abattoir #2), in Antananarivo city, central Madagascar. The only period when Abattoir #1 was not surveyed was during 15 days in September 2013. The dataset included 41,178 animals of local African breed and 27,254 pigs of exotic breed. The animals were raised in 79 different communes distributed in 36 districts belonging to 14 regions (out of a total of 22 administrative regions in Madagascar). On average, 5702 animals were slaughtered monthly; a maximum of 8028 animals slaughtered was reported for December, reflecting the increased demand for pork for New Year celebrations (Fig. 1).

Post-mortem inspection identified 3219 pigs with cysticercosis lesions. The overall apparent prevalence was estimated at 4.6 % [4.2 – 5.0 %]. The corrected overall prevalence - defined as the estimation of the prevalence after taking into account the sensitivity of meat inspection - was 21.03 % [19.18- 22.87 %].

### Differences between geoclimatic regions

Combined investigations of the central, western and northern geoclimatic regions revealed contrasted situations. Differences based on the geoclimatic divisions were analyzed for local indigenous pigs only, given the poor availability of information about commercial breed pigs raised in the western and northern regions. A total of 1709 *T. solium* cysticercosis positive pigs and 24,951 cysticercosis free local pigs from the central area (6.4 %), 179 *T. solium* positive and 2182 *T. solium* free animals from the western area (7.6 %), and, 983 *T. solium* positive and 9439 *T. solium* free pigs from the northern area (9.4 %) were recorded. Taking into account the effect of the season, the estimated prevalences of *T. solium* positive carcasses (Table 1) varied little between regions: 7.9 % [6.0 – 9.9 %] in the northern and western regions, and 7.3 % [6.0-8.6 %] in the central region. However, local pigs from the central region were as likely to be as infected as those in the western region (OR = 1.11 [0.9-1.4]; n.s.), but less infected than those in the northern region (OR = 1.45 [1.3-1.6]; p-value <0.001), revealing that fattened pigs originating from the northern region and slaughtered in Antananarivo were significantly more at risk of *T. solium* cysticercosis than pigs raised in the central region. This result should be interpreted considering the season and the slaughterhouse where the surveillance was conducted: in particular, Table 1 shows that, (i) at Abattoir#2, carcasses of local pigs raised in the northern region were less frequently infected than pigs produced in the central region (OR = 0.34 [0.25-0.47]; p-value <0.001); (ii) fall was an at-risk period for pigsproduced in the western region, and spring was an at-risk period for pigsproduced in the northern region, compared with pig carcasses produced in the central region and slaughtered in summer.

**Table 1:** Multiple regression analysis of the association between different individual parameters and porcine cysticercosis infection^a^ in pigs (local breed only) in the central, northern and western climatic regions of Madagascar (n = 39,443)

Variable	Level	Infected (n = 2871)	Not infected (n = 36,572)	Adjusted odds ratio (95 % CI)^c^	*p*-value
		n (%)^b^	n (%)^b^		
Season	Fall	399 (6.3 %)	5940 (93.7 %)	0.64 (0.56-0.75)	<10^-3^
	Winter	781 (6.4 %)	11,354 (93.6 %)	0.70 (0.62-0.79)	<10^-3^
	Spring	321 (7.1 %)	4181 (92.9 %)	0.76 (0.65-0.89)	<10^-3^
	Summer	1370 (8.3 %)	15,097 (91.7 %)	Reference	
Abattoir	Abattoir2	248 (7.9 %)	3334 (92.1 %)	1.48 (1.20-1.83)	<10^-3^
	Abattoir1	2623 (7.3 %)	33,238 (92.7 %)	Reference	
Region	West	179 (7.6 %)	2182 (92.4 %)	1.13 (0.91-1.4)	N.S
	North	983 (9.4 %)	9439 (90.6 %)	1.46 (1.29-1.66)	<10^-3^
	Central	1709 (6.4 %)	24,951 (93.6 %)	Reference	

^a^Determined using veterinary visual inspection at the slaughterhouse

^b^(%) refers to row percentages

^c^ *CI* Confidence interval

Among local pigs produced in the southern region and traded in Antananarivo, 119 positive animals were detected vs. 1616 negative animals (6.86 %). The estimated apparent prevalence of *T. solium* in pork carcasses was 6.2 % [4.7 – 7.8 %], and did not vary with the season. However, differences were observed between the two abattoirs: abattoir #2 (OR = 0.7 [0.47 - 1.04]; p-value < 0.001) received fewer infected pigs from the southern region than abattoir#1, independently of the season.

**Fig. 2:**
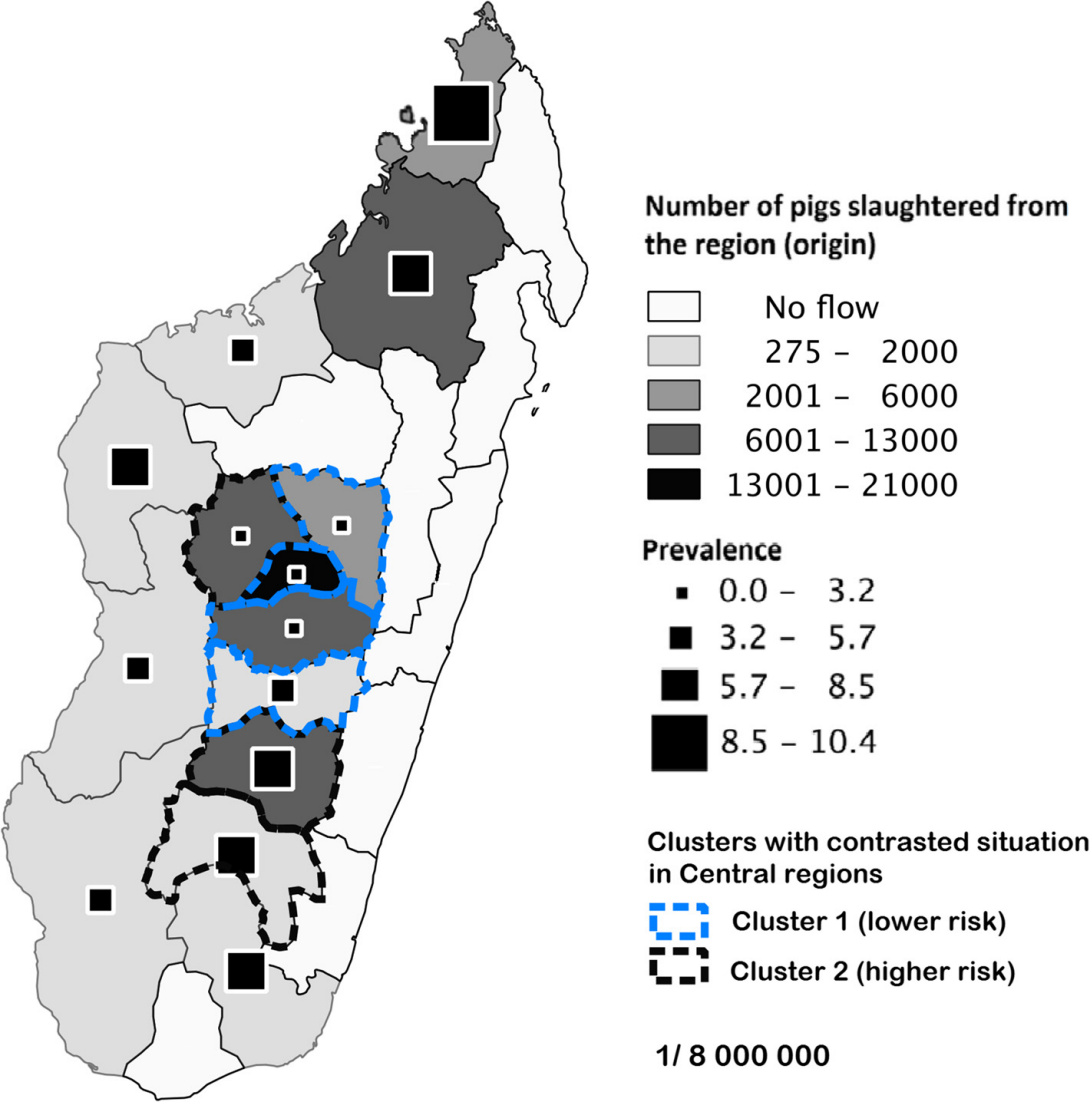
Apparent prevalence of porcine cysticercosis based on the detection of cysticercosis lesions during post-mortem inspection of 68,432 pigs slaughtered in 2 urban abattoirs in Antananarivo (Madagascar) from March 2013 to February 2014. The size of the black squares indicates the prevalence, while the shade of grey indicates the number of pigs slaughtered according to their region of origin. Dashed lines identified the clusters of low (blue) and high (black) risk for porcine cysticercosis for central regions only

The seven administrative central upland regions were investigated separately (Table 2) taking three factors into account: the breed (indigenous vs. commercial breed), season, and abattoir; 1884 positive and 50,114 negative animals were recorded (3.62 %). The estimated apparent prevalence in the central region was 3.8 % [2.9-4.6 %], independently of the breed, the season or the abattoir concerned. Nevertheless, two clusters were identified, revealing contrasted situations among the central regions (see Fig. 2): Cluster 1 grouped four regions with low prevalence and with no significant differences among them: Itasy (2.5 % [0.9-4.1 %]), Vakinankaratra (3.3 % [1.9-4.8 %]), Analamanga (3.6 % [2.0-5.2]), and Amoroni’Mania (4.4 % [2.5-6.3 %]); Cluster 2 grouped three regions located farther from the capital than the regions grouped in Cluster 1, with a significantly higher prevalence. The regions grouped in Cluster 2 were identified as at-risk production areas for the final market in the Malagasy capital: the highest estimated prevalence (7.1 % [5.9-8.4 %]) was found in Ihorombe around Fianarantsoa city (OR: 1.60 [1.20-2.13]; p-value < 0.001); prevalences in Haute Matsiatra and in Bongolava were estimated at 5.3 % [3.0-7.5 %] (OR: 1.63 [1.45-1.84]; p-value < 0.001), and 4.1 % [2.1-6.0 %] (OR: 1.21 [1.02-1.43]; p-value < 0.01) respectively.

**Table 2:** Multiple regression analysis of the association between individual (breed) and grouped (abattoir, season) parameters and porcine cysticercosis infection^a^ in pigs from 7 regions in the central upland region of Madagascar (n = 51998)

Risk factor	Category	Infected (n = 1884)	Not infected (n = 50114)	Adjusted odds ratio (95 % CI)^c^	*p*-value
		n (%)^b^	n (%)^b^		
Breed	indigenous	1709 (6.4 %)	24,951 (93.6 %)	8.46 (6.71-10.7)	<10^-3^
	Commercial	175 (0.7 %)	25,163 (99.3 %)	Reference	
Abattoir	Abattoir2	170 (4.5 %)	3578 (95.5 %)	3.6 (2.5-5.2)	<10^-3^
	Abattoir1	1714 (3.6 %)	46,536 (96.4 %)	Reference	
Season	Fall	271 (3.1 %)	8529 (96.9 %)	0.53 (0.32-0.89)	<10^-2^
	Winter	490 (3.3 %)	14,426 (96.7 %)	0.37 (0.23-0.6)	<10^-3^
	Spring	210 (3.6 %)	5676 (96.4 %)	0.32 (0.17-0.61)	<10^-3^
	Summer	913 (4.1 %)	21,483 (95.9 %)	Reference	
Region	Amoron’I Mania	53 (5.7 %)	870 (94.3 %)	1.17 (0.87-1.58)	N.S
	Analamanga	53 (2.1 %)	2435 (97.9 %)	1.1 (0.79-1.52)	N.S
	Vakinankaratra	129 (1.5 %)	8481 (98.5 %)	0.1 (0.8-1.2)	N.S
	Bongolava	199 (3.2 %)	6034 (96.8 %)	1.2 (1.02-1.44)	<10^-2^
	Haute Matsiatra	937 (7.4 %)	11,763 (92.6 %)	1.64 (1.45-1.84)	<10^-3^
	Ihorombe	58 (8.1 %)	659 (91.9 %)	1.60 (1.2-2.1)	<10^-3^
	Itasy	455 (2.2 %)	19,872 (97.8 %)	Reference	

^a^Determined using visual meat inspection at the slaughterhouse

^b^(%) refers to row percentages

^c^ *CI* Confidence interval

### Risk factors

In the seven administrative regions of the central upland considered in this study, multivariate logistic regression analyses (Table 2) showed that both the breed and the season influenced apparent prevalence. First, indigenous local pigs were 8.5 times [6.7 – 10.7] more likely to be infected than exotic improved pigs. In addition, urban consumers who lived in Antananarivo and ate pork produced in the central region were more likely to find cysticercosis in pork during the rainy season (austral summer), in particular during religious celebrations in December, than in other periods. Veterinary inspections reported less cysticerci in pig carcasses in the cool dry season (April and May) (OR = 0.53 [0.32-0.88]; p-value < 0.01), winter/cold dry season (June to August) (OR = 0.37 [0.23 – 0.60]; p-value < 0.001) or spring/warm dry season (September and October) (OR = 0.32 [0.16 – 0.61]; p-value < 0.001) than in summer/warm rainy season (November to March). Finally, pigs raised in the central region also gave different results with respect to the two abattoirs: animals slaughtered in Abattoir#2 were 3.6 times [2.5 – 5.2] more likely to be infected than in the Abattoir#1.

## Discussion

Our main result confirmed that the burden of pork contaminated by *T. solium* is underestimated not only by official statistics, which cite 0.5 to 1 % prevalence [2], but also by a preliminary assessment of the disease burden in four Antananarivo slaughterhouses in 2010 [19]. Moreover, the overall prevalence rate of *T. solium* cysticercosis, albeit after correction, appears to be higher than rates reported in other African countries, e.g. 14.4 % in Nigeria [20] or 15.7 % in Cameroon [21].

Malagasy indigenous pigs were 8.5 times [6.7 – 10.7] more likely to be infected than exotic improved pigs, as also observed to a lesser extent in India [22], and Zambia [23]. In contrast, commercial or crossbred animals were particularly mentioned as a risk factor for cysticercosis in Zambia [24] and South Africa [25]. This is more likely an effect of the farming system in which the animals were raised than to a hypothetical genetic resistance of the exotic pig breeds. Commercial pigs, e.g. Large White or Landrace breeds, in the central regions of Madagascar are mostly raised in confined conditions and only indigenous pigs roam and scavenge freely [26]; consequently local pigs are more at risk of ingesting taenid eggs from human faeces. However, in a recent study in Tanzania [27], confining pigs in rural areas as a way of preventing porcine cysticercosis was called into question as confining the animals all year round failed to reduce the seroprevalence of porcine cysticercosis, suggesting that pigs were either not fully confined or that infection occurred in the pens, and hence environmental contamination by Taenia eggs within and around farms. Consequently, surveillance of the environment in and around farms where pigs are raised in confined conditions should not be neglected.

The number of cases observed in urban slaughterhouses increased during austral summer (rainy season). In Burkina Faso, a similar increase during the rainy season was associated with pig rearing practices. When farmers are temporarily short of pig feed or their income is reduced, they allow their pigs to roam [28]. In Madagascar, the answer is probably the organization of the trade sector during periods of underproduction: during the rainy season, i.e. when the Christmas and New Year festivities approach, the normal supply chain of live pigs is unable to provide enough meat to satisfy the increased demand for pork, and, as the prices rise, traders may be less selective and accept pigs independently of their health status. Whereas the pork trade has been said to influence the transmission of the parasite [29], it is its efficacy in limiting the circulation of infected animals to the final market that may be at fault.

A high but spatially heterogeneous prevalence of the parasite was revealed among the pig population. However, the overall apparent prevalence may be underestimated, in particular for distant regions. As our study only used data from urban slaughterhouses (i.e. at the final market) it does not give a true picture of the disease in coastal regions, either on commercial farms or on smallholder pig farms. First, there is a local trade sector in every region, and commercial pig farms preferentially supply local demand in secondary cities. Then, the highly infected carcasses detected by lingual palpation at live pig markets are usually diverted toward the informal market [8, 30], for local consumption in street restaurants or for processing of low-cost pork products [2]. In this way, these animals escape any existing surveillance system or control measures. Transversal studies are thus needed in rural and coastal regions and in peri-urban production areas close to secondary cities to complete our epidemiological information.

With our surveillance system, light infections may be missed. Because previous studies underlined the lack of sensitivity of meat inspection for *T. solium* detection, especially in developing countries in Africa, which may lead to underestimation of the number of pig carcasses harboring cysts [24, 31, 32], we accounted for this error by using a detection fraction to estimate the corrected overall prevalence [12]. In addition, we maximized the size of our survey sample in order to be as representative as possible of the total pig production sold in the Malagasy capital city: Hence, based on the carcass of one pig (60 kg on average, personal field observations), the survey dataset (n = 68,432) corresponded to 63 % of the total annual demand in Antananarivo city, which has a population of 1.23 million [33], and where 5.3 kg of pork is consumed per inhabitant per year [34, 35].

However, the cysticerci stages, i.e. viable or degenerated, were not recorded, leading to potential overestimation of the risk for consumers. The true risk of contracting a tapeworm by eating pork may be difficult to evaluate in the absence of reliable data either about the food behaviours of Malagasy consumers or about the organization of the pork value chain.

## Conclusion

In conclusion, we recommend: (1) increasing the detection sensitivity of meat inspection, particularly by improving surveillance in urban abattoirs during austral summer, especially in December and January, and by targeting pigs of indigenous breeds raised in northern and southern regions; (2) conducting surveys and risk assessment studies, firstly at farm level to investigate the distribution of porcine cysticercosis in endemic regions, i.e. disease mapping and cluster analysis, and secondly at value chain level to better understand the informal trade networks for live pigs and pork to better identify at-risk networks or market places, in order to implement efficient prevention and control measures; and (3) raising the awareness of farmers and stakeholders by promoting evidence-based health education as a specific control measure.

## References

[1] Maurice J (2014). Of pigs and people? WHO prepares to battle cysticercosis. Lancet.

[2] Rasamoelina-Andriamanivo H, Porphyre V, Jambou R (2013). Control of cysticercosis in Madagascar: beware of the pitfalls. Trends Parasitol.

[3] Andriantsimahavandy A, Ravaoalimalala VE, Rajaonarison P, Ravoniarimbinina P, Rakotondrazaka M, Raharilaza N (2003). Situation épidémiologique actuelle de la cysticercose à Madagascar. Arch Inst Pasteur de Madagascar.

[4] Michelet L, Carod J-F, Rakotondrazaka M, Ma L, Gay F, Dauga C (2010). The pig tapeworm *Taenia solium*, the cause of cysticercosis: Biogeographic (temporal and spacial) origins in Madagascar. Mol Phylogenet Evol.

[5] Costard S, Porphyre V, Messad S, Rakotondrahanta S, Vidon H, Roger F (2009). Multivariate analysis of management and biosecurity practices in smallholder pig farms in Madagascar. Prev Vet Med.

[6] Goussanou S, Kpodekon T, Saegerman C, Azagoun E, Youssao A, Farougou S (2013). Spatial distribution and risks factors of porcine cysticercosis in southern Benin based meat inspection records. Int J Microbiol Res.

[7] Phiri IK, Dorny P, Gabriel S, Willingham Iii AL, Speybroeck N, Vercruysse J (2002). The prevalence of porcine cysticercosis in Eastern and Southern provinces of Zambia. Vet Parasitol.

[8] Phiri IK, Dorny P, Gabriel S, Willingham AL, Sikasunge C, Siziya S (2006). Assessment of routine inspection methods for porcine cysticercosis in Zambian village pigs. J Helminthol.

[9] Boa ME, Kassuku AA, Willingham Iii AL, Keyyu JD, Phiri IK, Nansen P (2002). Distribution and density of cysticerci of *Taenia solium* by muscle groups and organs in naturally infected local finished pigs in Tanzania. Vet Parasitol.

[10] Rakotoharinome M, Randriamparany T, Pognon D, Chane Ming J, Idoumbin JP, Cardinale E (2014). Prevalence of antimicrobial residues in pork meat in Madagascar. Trop Anim Health Prod.

[11] Porphyre V, Rakotoharinome M, Pognon D, Randriamparany T, Prévost S, Le Bizec B (2013). Residues of medroxyprogesterone acetate detected in sows at slaughterhouse, Madagascar. Food Addit Contam Part A.

[12] Dupuy C, Morlot C, Gilot-Fromont E, Mas M, Grandmontagne C, Gilli-Dunoyer P (2014). Prevalence of *Taenia saginata* cysticercosis in French cattle in 2010. Vet Parasitol.

[13] Dorny P, Phiri IK, Vercruysse J, Gabriel S, Willingham Iii AL, Brandt J (2004). A Bayesian approach for estimating values for prevalence and diagnostic test characteristics of porcine cysticercosis. Int J Parasit.

[14] Hosmer DW, Lemeshow S. Applied logistic regression. John Wiley & Sons, Inc., Hoboken, New Jersey, USA.

[15] Akaike H (1974). A new look at the statistical model identification. IEEE T Automat Contr.

[16] Hosmer DW, Lemeshow S. Applied logistic regression. New York; 2000.

[17] R Development Core Team. R: A language and environment for statistical computing. In http://www.r-project.org/; Vienna. 2008.

[18] QGIS (2012). Development team: QGIS Geographic Information System (Open Source Geospatial Foundation Project).

[19] Porphyre V, Betson M, Rabezanahary H, Mboussou Y, Zafindraibe NJ, Andriamanivo H, et al. *Taenia solium* porcine cysticercosis in Madagascar: comparison of immuno-diagnostic techniques and estimation of the prevalence in pork carcasses traded in Antananarivo city (accepted). Vet Parasitol. 2015, X:XXX-XXX. 10.1016/j.vetpar.2015.08.027 26342625

[20] Gweba M, Faleke OO, Junaidu AU, Fabiyi JP, Fajinmi AO (2010). Some risk factors for *Taenia solium* cysticercosis in semi-intensively raised pigs in Zuru, Nigeria. Vet Ital.

[21] Assana E, Zoli A, Sadou HA, Nguekam, Vondou L, Pouedet MSR, et al. Prevalence of porcine cysticercosis in Mayo-Danay (North Cameroon) and Mayo-Kebbi (Southwest Chad) [French]. Revue Elev Méd vét Pays Trop. 2001, 54:123-127.

[22] Mohan VR, Tharmalingam J, Muliyil J, Oommen A, Dorny P, Vercruysse J (2013). Prevalence of porcine cysticercosis in Vellore, South India. Trans Roy Soc Trop Med Hyg.

[23] Sikasunge CS, Phiri IK, Phiri AM, Dorny P, Siziya S, Willingham Iii AL (2007). Risk factors associated with porcine cysticercosis in selected districts of Eastern and Southern provinces of Zambia. Vet Parasitol.

[24] Sikasunge CS, Phiri IK, Phiri AM, Siziya S, Dorny P, Willingham Iii AL (2008). Prevalence of *Taenia solium* porcine cysticercosis in the Eastern, Southern and Western provinces of Zambia. Vet J.

[25] Krecek RC, Mohammed H, Michael LM, Schantz PM, Ntanjana L, Morey L (2012). Risk factors of porcine cysticercosis in the Eastern Cape Province, South Africa. PLoS One.

[26] Madec F, Hurnik D, Porphyre V, Cardinale E. Good practices for biosecurity in the pig sector – Issues and options in developing and transition countries. Rome: FAO; 2010, 74 http://www.fao.org/3/a-i1435e.pdf.

[27] Braae U, Magnussen P, Lekule F, Harrison W, Johansen M. Temporal fluctuations in the sero-prevalence of *Taenia solium* cysticercosis in pigs in Mbeya Region, Tanzania. Parasit Vectors. 2014, 7. 10.1186/s13071-014-0574-7 PMC4266939 25471610

[28] Ganaba R, Praet N, Carabin H, Millogo A, Tarnagda Z, Dorny P (2011). Factors associated with the prevalence of circulating antigens to porcine cysticercosis in three villages of Burkina Faso. PLoS Negl Trop Dis.

[29] Praet N, Kanobana K, Kabwe C, Maketa V, Lukanu P, Lutumba P (2010). *Taenia solium* cysticercosis in the Democratic Republic of Congo: how does pork trade affect the transmission of the parasite?. PLoS Negl Trop Dis.

[30] da Silva MRM, Uyhara CNS, Silva FH, Espindola NM, Poleti MD, Vaz AJ (2012). Cysticercosis in experimentally and naturally infected pigs: parasitological and immunological diagnosis. Pesquisa Vet Brasil.

[31] Dupuy C, Morignat E, Maugey X, Vinard J-L, Hendrikx P, Ducrot C (2013). Defining syndromes using cattle meat inspection data for syndromic surveillance purposes: a statistical approach with the 2005-2010 data from ten French slaughterhouses. BMC Vet Res.

[32] Nsadha Z, Kawuma P, Doble L, Kivali V, Ojok FEL, Nasinyama G (2014). Diagnostic efficiency of meat inspection service to detect *Taenia solium* cysticercostic pork at Wambizi pig abattoir, Kampala, Uganda: Implications for public health. Biomed Sci.

[33] INSTAT Madagascar: Human population census in Madagascar 1993-2011. http://www.instat.mg. In Book Human population census in Madagascar 1993-2011*.* http://www.instat.mg (Editor ed.^eds.). City; 2011.

[34] Ramamaonjisoa S, Randrianasolo SA, Rafolo R. Etude des aspects socioculturels de l'extension et de la consommation du porc à Madagascar. In Book Etude des aspects socioculturels de l'extension et de la consommation du porc à Madagascar (Editor ed.^eds.). pp. 168. City: Comité de coordination des deuxièmes journées de la filière porcine de l'Océan Indien; 1998:168.

[35] Andriamparany HM. Evaluation des impacts économiques des maladies porcines importantes à Madagascar. Faculté de Médecine. Université d’Antananarivo, Thèse de Médecine Vétérinaire; Antananarivo, Madagascar 2012.

